# Uncovering Candidate Genes Controlling Major Fruit-Related Traits in Pepper *via* Genotype-by-Sequencing Based QTL Mapping and Genome-Wide Association Study

**DOI:** 10.3389/fpls.2020.01100

**Published:** 2020-07-23

**Authors:** Hea-Young Lee, Na-Young Ro, Abhinandan Patil, Joung-Ho Lee, Jin-Kyung Kwon, Byoung-Cheorl Kang

**Affiliations:** ^1^ Department of Plant Science, Plant Genomics and Breeding Institute and Vegetable Breeding Research Center, College of Agriculture and Life Sciences, Seoul National University, Seoul, South Korea; ^2^ National Academy of Agricultural Science, National Agrobiodiversity Center, Rural Development Administration, Jeonju, South Korea

**Keywords:** *Capsicum*, domestication, fruit-related traits, genotype-by-sequencing, genome-wide association study, quantitative trait locus, linkage disequilibrium

## Abstract

All modern pepper accessions are products of the domestication of wild *Capsicum* species. However, due to the limited availability of genome-wide association study (GWAS) data and selection signatures for various traits, domestication-related genes have not been identified in pepper. Here, to address this problem, we obtained data for major fruit-related domestication traits (fruit length, width, weight, pericarp thickness, and fruit position) using a highly diverse panel of 351 pepper accessions representing the worldwide *Capsicum* germplasm. Using a genotype-by-sequencing (GBS) method, we developed 187,966 genome-wide high-quality SNP markers across 230 C*. annuum* accessions. Linkage disequilibrium (LD) analysis revealed that the average length of the LD blocks was 149 kb. Using GWAS, we identified 111 genes that were linked to 64 significant LD blocks. We cross-validated the GWAS results using 17 fruit-related QTLs and identified 16 causal genes thought to be associated with fruit morphology-related domestication traits, with molecular functions such as cell division and expansion. The significant LD blocks and candidate genes identified in this study provide unique molecular footprints for deciphering the domestication history of *Capsicum*. Further functional validation of these candidate genes should accelerate the cloning of genes for major fruit-related traits in pepper.

## Introduction

Pepper (*Capsicum* species), like other Solanaceae family members including tomato and potato, is a New World genus with a primary center of diversity in Bolivia and Peru (Nee et al., 2006). *Capsicum* comprises more than 30 species, and the domestication of five of these species in the Americas, including the economically important plants *C. annuum*, *C. baccatum*, *C. chinense*, *C. frutescens*, and *C. pubescens*, dates back to 6,000 BC (Moscone et al., 2007; Paran and Van Der Knaap, 2007; Cheng et al., 2016). Peppers are referred to as capsicum, pimento, sweet pepper, red pepper, cayenne pepper, bird’s eye pepper, jalapenos, or habaneros based on fruit shape and pungency (Moscone et al., 2007; Babu et al., 2011), and have various uses as vegetables, seasonings, ornamental plants, and medicinal crops. The easy cultivation of pepper has led to their widespread use worldwide, especially in tropical regions (Moscone et al., 2007; Babu et al., 2011). The majority of wild forms of *Capsicum* spp. display perennial herbaceous growth, with a small, erect, deciduous growth habit and red, pungent, soft-fleshed fruits (Paran and Van Der Knaap, 2007).

Among Solanaceae species, domestication-related traits have been described for tomato (Bai and Lindhout, 2007; Giovannoni 2018), potato (Li et al., 2018), and eggplant (Doganlar et al., 2002; Meyer et al., 2012). These traits are generally referred to as “domestication syndrome” because they can be used to distinguish cultivated crops from their progenitors (Doebley et al., 2006). The domestication syndrome traits are not fully elucidated for pepper. During domestication, *Capsicum* spp. might have been selected for fruit morphology and pungency (Babu et al., 2011; Che and Zhang, 2019). Other pepper domestication traits include a non-deciduous habit, fruit that remains on the plant until harvest, and pendent fruit orientation (Kaiser, 1935 Paran and Van Der Knaap, 2007). However, underlying genes are largely known.

Genetic and genomic analyses of cultivated crops and wild relatives have provided evidence for domestication by revealing selection footprints in the key genes controlling domestication traits (Zeder et al., 2006; Stitzer and Ross-Ibarra, 2018). Recent genetic and archaeological studies have revealed the spatiotemporal origins and processes underlying the domestication of these traits and have allowed domestication traits to be divided into two types based on the underlying genes. Some domestication traits are controlled by genes called ‘domestication genes’ that were subjected to early selection of major-effect QTLs, while other traits are controlled by genes that were selected later to produce diversified, improved crops; these genes are called ‘improvement genes’ (Pickersgill, 2007). Wang and Bosland (2006) published a comprehensive summary of genetic studies on *Capsicum* genes performed from 1912 to 2006 that lists 292 genes for various traits in pepper, including morphological and physiological traits, male sterility, and resistance to nematodes, diseases, and herbicides.

Most traditional QTL analyses in pepper have focused on fruit morphology-related traits. These studies have involved low-throughput genotyping or focused only on identifying the genes governing these traits. For instance, genetic mapping studies identified QTLs for fruit length, fruit width (FWd), fruit weight (FWg), pericarp thickness (PT), and fruit position (FP). Among these, *fs2.1*, *FrSHP2.1*, and *fs3.1* are the major QTLs for fruit shape; these QTLs are located on chromosomes 2 and 3 (Chaim et al., 2001; Chaim et al., 2003; Rao et al., 2003; Zygier et al., 2005; Barchi et al., 2009; Borovsky and Paran, 2011; Mimura et al., 2012; Hill et al., 2017; Chunthawodtiporn et al., 2018).

By contrast, due to advancements in next-generation sequencing (NGS) techniques and the availability of newer populations of tomato, six representative gene families were identified to control fruit size in this crop, including the Cell Number Regulator (CNR), Cytochrome P450 A78 class (CYP78A), IQ domain, Ovate Family Protein (OFP), YABBY, and WOX gene families (Monforte et al., 2014; Lin et al., 2014; Sacco et al., 2015; Soyk et al., 2017). Candidate genes belonging to these families such as *CNR*, *SlKLUH*, *SUN*, *OVATE*, *FAS*, and *LC* have been cloned, and their roles in regulating fruit elongation, locule number, and fruit shape have been well characterized (Xiao et al., 2008; Guo and Simmons, 2011; Rodriguez et al., 2011; Chakrabarti et al., 2013). These findings from tomato were successfully utilized for QTL mapping and downstream gene analysis in pepper, shedding light on the complex genetic architecture and genomic regions that govern these quantitatively inherited traits (Ramchiary et al., 2014; Wang et al., 2015; Chunthawodtiporn et al., 2018; Colonna et al., 2019).

Since the release of the first reference genome of pepper (Kim et al., 2014), genome-wide association study (GWAS) has been used to analyze only a few traits in pepper such as fruit weight (Nimmakayala et al., 2016a), capsaicinoid contents (Nimmakayala et al., 2016a; Han et al., 2018), peduncle length (Nimmakayala et al., 2016b), and fruit size and shape (Colonna et al., 2019) using diverse pepper germplasms. Combined QTL mapping and GWAS has been utilized to avoid identifying false-positive QTLs or associations for major fruit-related traits in pepper.

The goals of the current study were to (1) explore the correlations among five important fruit-related traits in pepper and (2) determine the significant genetic regions or genes governing genetic variations in the major fruit-related traits with strong evidence for selection during domestication. We obtained high-quality SNPs *via* genotype-by-sequencing (GBS) and subjected them to GWAS. We obtained candidate genes underlying the QTLs detected by GWAS and characterized their functions, laying the foundation for further functional validation and cloning of candidate genes for major fruit-related traits in pepper.

## Materials and Methods

### Plant Materials

A collection of 351 *Capsicum* accessions known as the ‘pepper GWAS population’ was used for analysis, comprising four major domesticated species including *C. annuum* (230 accessions), *C. baccatum* (48 accessions), *C. chinense* (48 accessions), and *C. frutescens* (25 accessions) (**Table S1**). Among the accessions in the pepper GWAS population, 250 were previously selected as a core set representing the genetic diversity of more than 4,600 accessions from 97 countries (Lee et al., 2016).

### Phenotypic Evaluation and Correlation Analysis

The pepper GWAS population was planted in a greenhouse at the Rural Development Administration (RDA)-Gene bank Jeonju, Republic of Korea (35°49′51.3” N, 127°03′47.1” E). Over a three-year period (2015–2017), six plants per accession were randomly planted, and the phenotypes of three plants per accession were evaluated. Five domestication traits were evaluated using a randomized block design, including four quantitative traits (fruit length [FL], width [FWd], weight [FWg], and pericarp thickness [PT]) and one qualitative trait (fruit position [FP]). All quantitative traits were measured using an electronic scale and a ruler. FP was scored as 1 to 3 (1 = erect, 2 = declining like a pendant, and 3 = intermediate). The correlation among the five traits was evaluated by Pearson correlation (r) analysis with SPSS software (IBM Corp. Released 2017. IBM SPSS Statistics for Windows, Version 25.0. Armonk, NY).

### Genomic DNA Extraction and GBS

Genomic DNA was extracted from the samples using the CTAB method (Lee et al., 2017; Siddique et al., 2019; Solomon et al., 2019) and diluted to 80 ng/µl in distilled water. GBS libraries were constructed *via* double digestion with two sets of restriction enzymes (*Pst*I/*Mse*I and *EcoR*I/*Mse*I) as previously described (Han et al., 2018; Siddique et al., 2019; Solomon et al., 2019). The digested DNA was ligated to adapters and amplified with ‘TA’ primers. The libraries were pooled in five tubes. The contents of the tubes were sequenced in separate lanes using the HiSeq 2000 platform (Illumina, San Diego, CA) at Macrogen (Seoul, Republic of Korea).

### Reference-Based SNP Calling and Construction of the SNP Set

Raw 101-bp reads from the libraries were trimmed to a minimum length of 80 bp and filtered to a quality score >Q30. The filtered reads were aligned to the *C. annuum* ‘CM334’ reference genome v.1.6, http://peppergenome.snu.ac.kr (Kim et al., 2017) using the Burrows-Wheeler Aligner program v.0.7.12 (Li and Durbin, 2010). For SNP calling and filtering, the GATK Unified Genotyper v.3.3-0 was used (Depristo et al., 2011). The SNP set was constructed using three steps: pre-filtering, imputation, and major filtering. First, pre-imputed SNPs were filtered to removed mono and tri-allelic SNP types and SNPs with a call rate >0.1. After pre-imputation filtering, SNPs with missing data were imputed using the FILLIN method in TASSEL (Bradbury et al., 2007). To obtain SNPs of suitable quality, hapSize was applied to obtain sequences ranging from 100 to 8,000 with two minSites (25, 50) and two minPres (250, 500). The final selected imputation option was dependent on the best option of regression (R^2^) values and the imputed ratio of minor and major alleles. Finally, the major filtering step was performed under the following conditions: minor allele frequency >0.05, SNP coverage >0.6, and inbreeding coefficient (IF) >0.8.

### Population Structure (Q) and Linkage Disequilibrium (LD) Estimations

To identify population stratification, principal component analysis (PCA) was performed using the ‘pcaMethods’ library in R software (Stacklies et al., 2007). The values of each PC were used as variables in the GWAS. The LD block of the GWAS population was estimated using PLINK v.1.9 (Chang et al., 2015) with the following settings: ‘–no-parents –no-sex –blocks no-pheno-req no-small-max-span –blocks-inform-frac 0.95 –blocks-max-kb 2000 –blocks-min-maf 0.05 –blocks-recomb-highci 0.9 –blocks-strong-highci 0.98 –blocks-strong-lowci 0.7’. The calculated LD block intervals were used to search for candidate genes for specific traits.

### Genome-Wide Association Study (GWAS) and Candidate Gene Identification

GWAS based on the compressed mixed linear model (CMLM) was conducted using the R package of Genomic Association and Prediction Integrated Tool (GAPIT) (Lipka et al., 2012) with the forward model selection using the Bayesian information criterion (BIC). The significance threshold −log_10_ *P*-value of the GWAS was determined using Bonferroni (1936) correction (FDR *P*-value  < 0.05) based on the number of independent SNPs in a population. The candidate genes inside LD regions with significant SNPs were investigated. Gene prediction was performed based on gff file v.2.0 of the CM334 v.1.6 reference genome (http://peppergenome.snu.ac.kr), and the function of each gene was predicted using Blast2GO (Götz et al., 2008) based on deduced protein sequences. To detect the physical positions of previous QTLs or orthologous genes from other species, BLAST searches of the pepper genome from the NCBI database (https://www.ncbi.nlm.nih.gov) were performed. To predict the molecular and biological functions of genes, the NCBI, Solanaceae (https://solgenomics.net), and *Arabidopsis* databases (https://www.arabidopsis.org) were used.

### QTL Mapping Using Recombinant Inbred Lines

To validate the GWAS results, recombinant inbred lines (RILs) were used for QTL mapping as described by Han et al. (2016). All information about the plant materials and phenotypes in this study were described in Han et al. (2016), but the genotyping results were altered using a more recent version of the reference genome (*C. annuum* ‘CM334’ v.1.6, http://peppergenome.snu.ac.kr). In brief, 120 F_7_–F_10_ RILs derived from a cross between pungent *C. annuum* ‘Perennial’ and non-pungent *C. annuum* ‘Dempsey’ were grown for 3 years (2011, 2012, and 2014) in two locations: Anseong (2011 and 2012a) and Suwon, Korea (2012b and 2014). After SNP calling, sequencing reads were aligned to *C. annuum* ‘CM334’ v.1.6. A modified sliding window approach was used to investigate recombination breakpoints and to construct a bin map of the RILs. Bins were used as markers to construct a genetic map using the Carthagene program (De Givry et al., 2005) with default threshold values. All detailed options were adapted from Han et al. (2016) and Siddique et al. (2019). Of the 18 reported traits (Han et al., 2016), we utilized major four fruit domestication-related traits (FL, FWd, FWg, and FP) for QTL analysis. Composite interval mapping (CIM) was performed with Windows QTL Cartographer 2.5 (Wang et al., 2012). The phenotypic values of each trait in the respective years and locations were analyzed separately to detect QTLs. The log of odds (LOD) threshold was determined by performing 1,000 permutation tests with 5% probability (P) for each trait, and the proportion of phenotypic variation (R^2^) for each QTL was estimated. The 95% confidence interval was used to represent the location of each QTL.

## Results

### SNP Discovery and Population Structure (Q) of a Pepper GWAS Population

**Figure 1:**
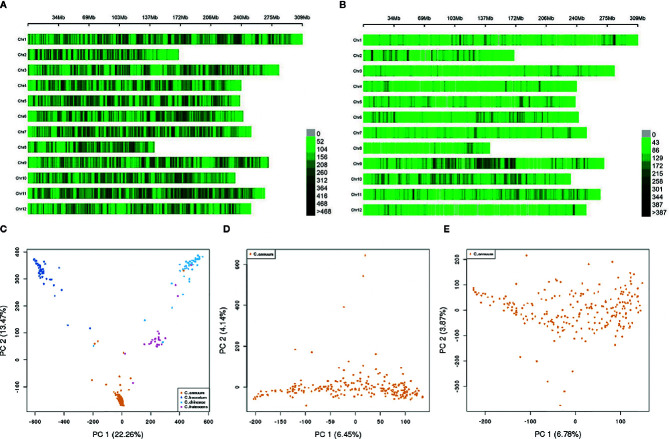
SNP distribution and population structure (Q) of the GWAS population. **(A, B)** SNP distribution across genotypes on 12 chromosomes within a 1 Mbp window size **(A)** SNP distribution across all accessions using 507,713 SNPs, **(B)** SNP distribution across all 230 C*. annuum* accessions using 187,966 SNPs). **(C–E)** Genetic distributions based on principal component analysis **(C)** Genetic distribution across all accessions using 507,713 SNPs, **(D)** Genetic distribution across 230 C*. annuum* accessions using 507,713 SNPs, **(E)** Genetic distribution across 230 C*. annuum* accessions using 187,966 SNPs).

We aligned sequences derived from GBS to the *C. annuum* cv. CM334 reference genome v. 1.6, http://peppergenome.snu.ac.kr (Kim et al., 2017). GBS genotyping of 351 accessions (**Table S1**) with two sets of libraries constructed using double digestion with two sets of restriction enzymes generated 8,717,361 SNPs. The GBS generated data is available in National Agricultural Biotechnology Information Center (NABIC, https://nabic.rda.go.kr/, ID= NV-0630-000001) Trimming and filtering-out of SNPs with a quality score <30, call rate <10%, and mono or tri-allelic SNPs types resulted in 1,869,524 SNPs (**Table S2**). To avoid potential errors in the interpretation of the GWAS results, the missing genotypes were imputed using the FILLIN method in the TASSEL package. Accordingly, approximately 26% of genotypes were imputed to minor alleles, and 21 and 59% of genotypes were imputed to hetero and major alleles, respectively, with a regression (R^2^) value of 0.82. Using this imputed genotype, the final filtering step was performed under the following conditions: MAF >0.05, SNP coverage >0.6, and IF >0.8. This step resulted in a set of 507,713 high-quality SNPs, which were evenly distributed on 12 chromosomes (**Figure 1A**, **Table S2**). Each SNP marker generated from this SNP set was named according to its physical position in the pepper reference genome.

As the genetic structure of a population can strongly affect the results of GWAS, we performed principal component analysis (PCA) to analyze population stratification. This analysis yielded four genetic clusters, with 22.26% (PC1) and 13.47% (PC2) of the genotypic variance in the first and second axes. Each cluster was well clustered by species, including *C. annuum*, *C. baccatum*, *C. chinense*, and *C. frutescens*. Although some accessions showed slight admixture, there were no conspicuous sub-clusters in the structure (**Figure 1C**).

### Phenotypic Diversity of Major Fruit-Related Domestication Traits in the GWAS Population

**Figure 2:**
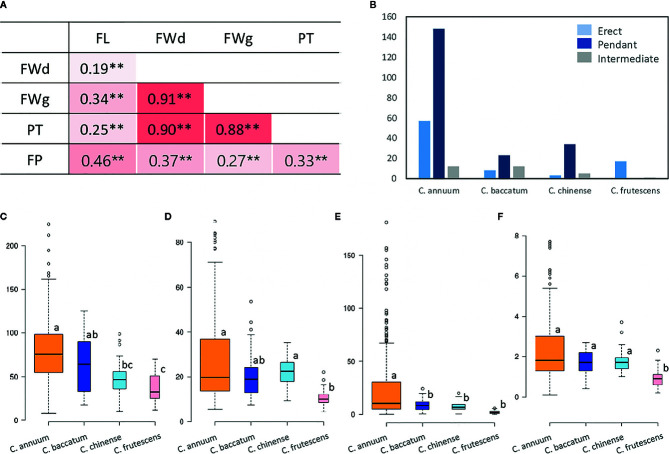
Phenotype performance and correlations among five major fruit-related domestication traits. **(A)** Pearson correlation coefficients (r) among the five investigated traits. Numbers indicate the correlations between two traits. Red blocks indicate positive correlations. Asterisks (**) represent a significant difference at *P*-value 0.01. **(B–F)** morphological distributions of four domestication-related traits among species (**B**: FP, **C**: FL, **D**: FWd, **E**: FWg, **F**: PT). Except FP, each box depicts the upper and lower quantile, with the median represented by a horizontal solid line. Outliers are indicated by dots. Different letters indicate significant difference at *P*-value <0.05, as determined by one-way ANOVA with Scheffe multiple comparison post-hoc test.

We evaluated 351 *Capsicum* accessions from four species with maximum genetic diversity (Lee et al., 2016) for three years to assess the range of phenotype variation of the five fruit-related domestication traits (FL, FWd, FWg, PT, and FP). We detected a consecutive reduction in the mean values of the quantitative traits during the three years of the experiment, except for FWd, which had a higher value in 2016 (25.1 mm) compared to 2015 (24.3 mm) and 2017 (23.7 mm). While the maximum average FL value (72.4 mm) was obtained in 2015, the minimum value (66.9 mm) was obtained in 2017. Similarly, FWg and PT showed the highest average values in 2015 (22.9 g and 2.5 mm, respectively), followed by 2016 (19.9 g and 2.0 mm, respectively) (**Table 1**). Three species showed either erect (40%) or pendant (60%) FP, whereas *C. frutescens* showed all erect FP. Intermediate FP was also observed in all species except *C. frutescens* (**Figure 2B**). High broad-sense heritability (H^2^) values were recorded for FL (0.8), FWd (0.81), FWg (0.83), and PT (0.72) (**Table 1**).

**Table 1:** Phenotypic variation of four quantitative traits identified by GWAS population-based analysis over a three-year period.

Traits	Unit	H^2^	2015				2016				2017			
			Min	Max	Mean	CV	Min	Max	Mean	CV	Min	Max	Mean	CV
FL	mm	0.80	7.0	316.0	72.4	54.4	8.9	255.0	70.0	52.8	8.9	200.9	66.9	50.4
FWd	mm	0.81	3.8	109.0	24.3	73.2	4.4	98.1	25.1	68.3	4.0	95.5	23.7	66.8
FWg	g	0.83	0.1	263.4	22.9	157.7	0.2	224.5	19.9	156.3	0.1	179.0	15.9	151.4
PT	mm	0.72	0.1	10.3	2.5	61.7	0.1	10.0	2.0	70.9	0.1	8.9	1.9	60.3

H^2^, broad-sense heritability; Min, minimum value; Max, maximum value; CV, coefficient of variation (%).

All five fruit-related domestication traits showed significant positive correlations (*P* = 0.01). Specifically, highly strong positive correlations were detected between FWg and FWd (r = 0.91), followed by FWd and PT (r = 0.90) and FWg and PT (r = 0.88). Although FL had slightly lower positive correlations with these three traits (FWd, FWg, PT), it was the most highly correlated with FP (r = 0.46) compared to the three other traits (**Figure 2A**). As the GWAS population was clustered by species, we performed ANOVA, which validated the variability in the traits among species (**Figures 2B–F**, **Tables S3** and **S4**). This analysis uncovered significant variation between species groups for the five traits, ranging from 9.63 to 22.68 (F, with *p* = 0.00), with a mean difference of 0.08 to 0.17 (η^2^), which also supported the differences among species (**Table S4**). Most of the traits showed the greatest mean values in the *C. annuum* accessions (FL: 78.8 mm, FWd: 27.6 mm, FWg: 26.8 g, PT: 2.4 mm), whereas the lowest mean values were detected in *C. frutescens* (**Figures 2C–F**).

### Linkage Disequilibrium (LD) Pattern and GWAS of the *C. annuum* Cluster

The minimize the confounding effect of interspecies variation and the corresponding false-positive errors, among the entire population set used in the experiment, we selected the *C. annuum* cluster, as it contained a sufficient number of accessions with high levels of phenotypic and genotypic diversity without any interrelated population stratification (**Figures 1D, E**). Since the five fruit-related traits showed high broad-sense heritability (H^2^) values (**Table 1**), indicating that genetic factors were the major determinants of the observed phenotypic variability, we subjected all traits to GWAS using the *C. annuum* CM334 v.1.6, http://peppergenome.snu.ac.kr reference genome.

In the *C. annuum* cluster, 187,966 high-quality SNPs were filtered for use in GWAS following the criteria described above (**Figure 1B**, **Table S2**). Using this SNP set, we compared the common and unique patterns of genetic variation in adjacent marker pairs of each chromosome by performing LD analysis throughout the genomes of the *C. annuum* accessions. We identified 12,234 LD blocks, with an average of 1,020 per chromosome. The average block size was 149 kb, each containing an average of 14 SNPs (**Table S5**). The LD blocks were named based on their order on each chromosome.

We detected high variation for all fruit-related traits over the three years of analysis except for the qualitative trait (FP), which was observed for only one year. Common SNPs that were consistently correlated for at least two years of investigation exceeding the significance threshold (−log_10_ *P >*6.575) were used to describe our results (**Figures S1** and **S2**, **Table S6**). Accordingly, a total of 178 common SNPs were identified, including 1 for FL, 148 for FWd, 28 for FWg, and 1 for PT. For FP, 52 significant SNPs from accessions with pendant, erect, and intermediate phenotypes were used for analysis (**Table S6**).

Of the 230 common SNPs, one SNP located on chromosome 4 (S04_227983120) was common both to FWg and PT. Furthermore, five SNPs on chromosome 9 (S09_100362495, S09_133144036, S09_136634514, S09_136634573, S09_143733895) and two SNPs on chromosome 12 (S12_14626660, S12_17471128) were detected for both FWd and FWg. Unlike these eight common SNPs, 222 SNPs were associated with 64 LD blocks distributed on chromosomes 2, 3, 4, 5, 6, 9, 10, and 12 (**Figure S3**, **Table S7**).

**Figure 3:**
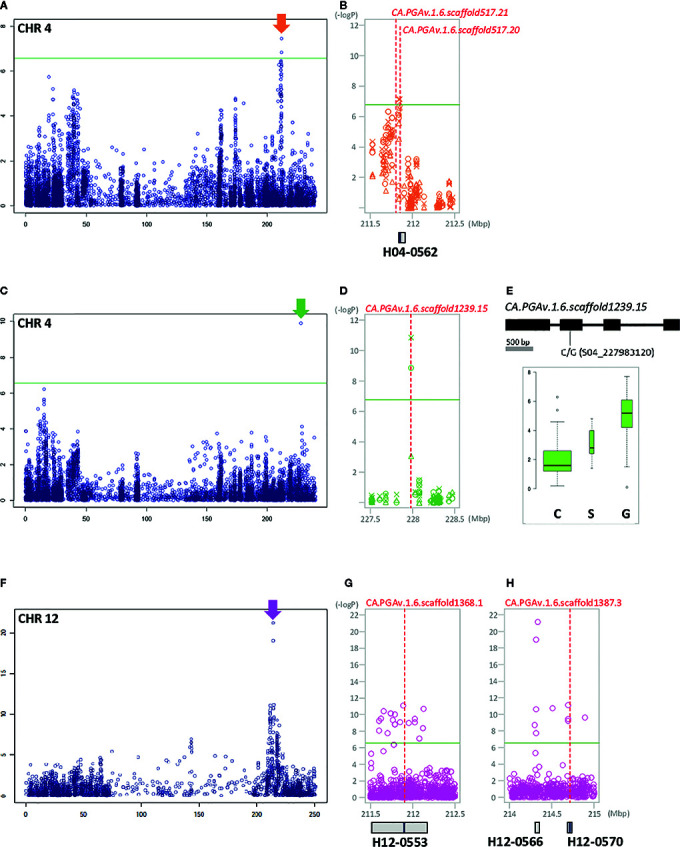
GWAS identifies significant SNPs and LD block regions containing genes controlling FL, PT, and FP in pepper. The left side of each Manhattan plot shows chromosome-wide associations. The most significant areas are indicated by arrows. The significant areas are shown in detail, with the three years of analysis represented by various shapes (circle: 2015, triangle: 2016, X: 2017). Under the Manhattan plot, LD blocks are represented by gray boxes. Candidate genes are indicated by red dotted lines, with the names and positions inside the LD block indicated by blue bars. **(A, B)**: FL, **(C–E)**: PT, **(F–H)**: FP, **(A)** association with FL on chromosome 4. **(B)** Close-up view of the significant LD block regions (211.5–212.5 Mbp) on chromosome 4. **(C)** Association with PT on chromosome 4. **(D)** Close-up view of the significant LD block regions (227.5–228.5 Mbp) on chromosome 4. **(E)** Gene structure with DNA polymorphism. Below the gene structure, boxplots show PT based on allelic differences of significant SNP; the width of each box is proportional to the square root of the number of accessions. **(F)** Association with FP on chromosome 12. **(G)** Close-up view of the significant LD block regions (211.5–212.5 Mbp) on chromosome 12. **(H)** close-up view of the significant LD block regions (214–215 Mbp) on chromosome 12.

**Figure 4:**
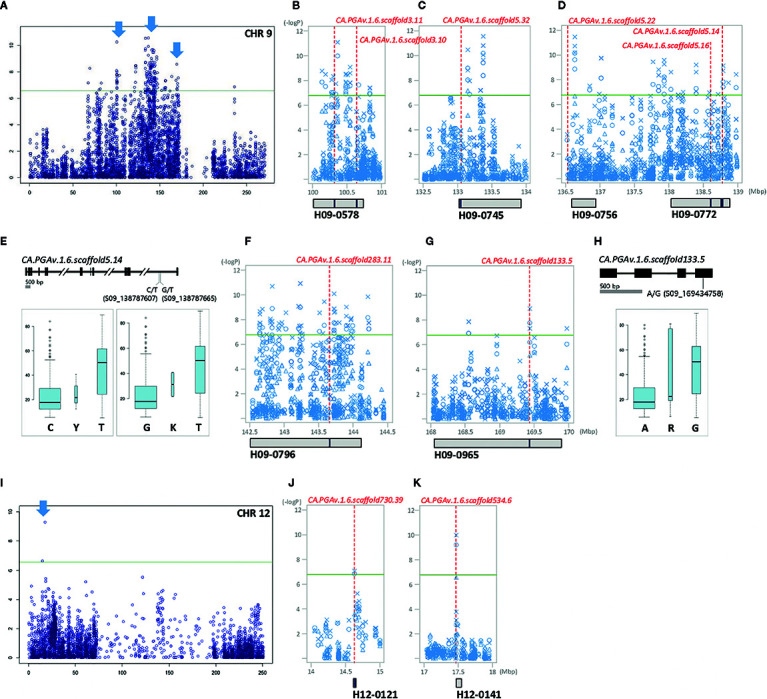
GWAS identifies significant LD block regions and correlated genes controlling FWd in pepper. The significant areas are shown in detail next to chromosome-wide Manhattan plots, in which all three years of analysis are represented by various shapes (circle: 2015, triangle: 2016, X: 2017). Under the Manhattan plot, LD blocks are represented by gray boxes. Candidate genes are represented by red dotted lines, with the names and positions indicated inside the LD blocks indicated by blue bars. **(A, I)**: chromosome-wide Manhattan plots. The most significant areas are indicated by arrows. **(B–D):** close-up views of the significant LD block regions (100–139 Mbp) on chromosome 9. **(E, H)**: gene structure with DNA polymorphism. Below the gene structure, boxplots of FWd based on allelic differences of significant SNPs are shown; the width of each box is proportional to the square root of the number of accessions. **(F, G)**: close-up views of the significant LD block regions (142.5–170 Mbp) on chromosome 9. **(J, K)**: close-up views of the significant LD block regions (14–18 Mbp) on chromosome 12.

In detail, for marker–trait associations per year, eight significant SNPs were identified for FL; of these, three SNPs (S03_19218749, S03_19218759, and S03_19254384) on chromosome 3 were detected only in 2017. Two SNPs were identified on chromosome 4, including S04_211838587 only in 2016 and S04_211848210 in all three years of the study. Two and one additional SNP (S05_12080328, S07_214135504, and S11_72669050) each on chromosomes 5, 7, and 11 were identified only in 2015 and 2017, respectively (**Figure S1**). SNP S04_211848210, which is associated with LD block H04-0562 on chromosome 4 in the region between 211.8 Mb and 211.9 Mb (spanning an interval of 64,916 bp), was expected to be highly correlated with FL, as it was consistently detected throughout the experiment (**Figures 3A, B**). For FWd, we detected 281 significant SNPs throughout the experimental period, with an average –log_10_ *P*-value ranging from 6 to 10.57 (**Figure S1**). Most of the significant SNPs (98.9%) were located on chromosome 9 from 67.4 to 171 Mb and were linked to 26 LD blocks (**Figure S3**). In 2015, nine unique significant SNP positions (eight on chromosome 9 and one on chromosome 7) were detected. There were 123 unique SNPs in 2016, all on the middle and distal regions of chromosome 9. While 146 common SNPs were detected in 2015 and 2016, only two SNPs were identified on chromosome 12. Notably, two SNPs (S09_133144036 and S09_136634573) associated with H09-0745 and H09-0756 were consistently identified in all three years of the study (**Figure 4**, **Table S6**).

**Figure 5:**
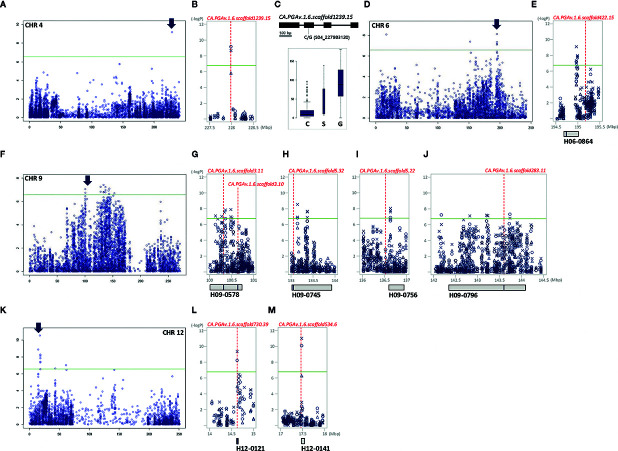
GWAS identifies significant LD block regions and correlated genes controlling FWg in pepper. **(A, D, F, K)**: chromosome-wide Manhattan plots. The most significant areas are indicated by arrows. The significant areas are shown in detail next to the chromosome-wide Manhattan plot, in which all three years of analysis are represented by various shapes (circle: 2015, triangle: 2016, X: 2017). Under the Manhattan plot, LD blocks are represented by gray boxes. Candidate genes are represented by red dotted lines with the names and positions indicated inside the LD blocks indicated by blue bars. **(B)** Close-up view of the significant LD block regions (227.5–228.5 Mbp) on chromosome 4. **(C)** Gene structure with DNA polymorphism. Below the gene structure, boxplots of FWg based on allelic differences of significant SNPs are shown; the width of each box is proportional to square root of the number of accessions. **(E)** close-up view of the significant LD block regions (194.5–195.5 Mbp) on chromosome 6. **(G–J):** close-up views of the significant LD block regions (100–144.5 Mbp) on chromosome 9. **(L, M):** close-up views of the significant LD block regions (14–18 Mbp) on chromosome 12.

Of the 101 significant SNPs detected for FWg, 58.4% were located on chromosome 9 (**Figures 5F–J**). Unlike the other traits examined in the study, significant SNPs for FWg were identified on all chromosomes except chromosomes 3 and 5. While chromosomes 1, 4, 7, and 8 contained one SNP each, chromosomes 2, 6, 10, 11, and 12 contained 3, 11, 9, 2, and 13 significant SNPs for this trait, respectively. Twenty-nine SNPs were commonly detected in at least two years on chromosomes 2, 4, 6, 9, 10, and 12. Seven unique SNPs on chromosomes 6, 7, 8, 9 10, and 12 were detected in 2015; 64 unique SNPs were detected on chromosomes 1, 6, 9, 10, 11, and 12 in 2016; and two unique SNPs were detected on chromosomes 10 and 11 in 2017 (**Figure S1**).

PT was associated with 9 SNPs, which were located in the 227, 197–199, 174, 12, and 62–243 Mbp regions of chromosomes 4, 6, 7, 11, and 12, respectively (**Figure S1**). Among these, seven SNPs (S06_197114855, S06_198980398, S06_199214893, S06_199214897, S07_174200667, S11_12782432, and S12_61862450) were detected only in 2015, S12_243181724 was detected only in 2017, and S04_227983120 was detected in both 2015 and 2016 (**Figures 3C–E**, **Figure S1**, **Table S6**).

A genome-wide association scan also revealed 52 significant SNPs associated with the variation in FP (**Table S6**). Most of the significant SNPs were located on chromosome 12, while three SNPs were detected in the 220 Mbp region on chromosome 3, and two SNPs were located at 161 and 198 Mbp on chromosome 5, respectively. Inside of chromosome 12, except for two SNPs detected in a 143.3 Mbp region, most significant SNPs were detected near the 211 to 219 Mbp region, with the highest association detected in the H12-0566 block area (**Figures 3F–H**).

### QTLs of Major Fruit-Related Domestication Traits in the RILs

To confirm the GWAS results, we examined QTLs for four major fruit-related domestication traits (FL, FWd, FWg, FP) using 120 RILs derived from a cross between *C. annuum* ‘Perennial’ and *C. annuum* ‘Dempsey’ (PDRIL); in these lines, 86 QTLs for 17 horticultural traits were previously mapped (Han et al., 2016). The only difference in the technique used in the current compared to the previous study is that here, we used the genetic map developed from the more recent version of the reference genome (CM334 v.1.6, http://peppergenome.snu.ac.kr). Based on the reference genome, we used 444,405 SNPs from 120 RILs and both parental lines to construct a binmap. Using a sliding window approach, all SNPs were grouped into 2,050 bins (**Figure S4**, **Table S8**). The average length of the bins was 0.55 Mb, ranging from 100 kb to 83.5 Mbp. The total genetic distance of the bin map was 1,123.6 cM (**Table S9**).

Using the same phenotypic information, a total of 17 QTLs were identified (**Table 2**). Each QTL was named based on an abbreviation of the trait name and the chromosome number following ‘PD_’. For each trait, three to five QTLs were detected, which were distributed throughout chromosomes 2, 3, 4, 5, 7, 9, 10, and 12. The phenotypic variation (R^2^) explained by each QTL ranged from 8.3% (*PD_FP9*) to 38.5% (*PD_FWd4*).

**Table 2:** QTLs controlling FL, FWd, FWg, and FP detected in PDRIL based on the CM334 v.1.6 reference genome.

Trait	QTL	Year	Chr.	Location (cM)	LOD	R^2^ (%)	Direction	Additive effect
FL	*PD_FL2*	2012b	2	34.8–36.8	5.2	15.0	+	2.0
	*PD_FL3.1*	2014	3	56.2–56.9	4.9	11.9	+	0.7
	*PD_FL3.2*	2011	3	60.1–62.1	5.1	13.1	+	0.7
	*PD_FL5*	2014	5	72–74	8.0	20.7	+	1.8
	*PD_FL9*	2012a	9	68.5–69.8	9.2	21.8	+	4.7
FWd	*PD_FWd3*	2014	3	86–89.6	3.7	10.1	−	0.2
	*PD_FWd4*	2012a	4	21.2–22.6	11.5	38.5	+	2.7
	*PD_FWd7.1*	2014	7	29.5–30.6	4.3	11.7	+	0.8
	*PD_FWd7.2*	2012b	7	29.6–30.6	5.4	13.8	+	0.8
FWg	*PD_FWg7.1*	2011, 2012a	7	29.6–30.3	8.4–11.8	19.7–30.2	+	12.4–13.1
	*PD_FWg7.2*	2012b	7	29.6–30.6	7.5	18.4	+	6.5
	*PD_FWg7.3*	2014	7	29.9–30.7	8.7	22.2	+	8.8
FP	*PD_FP9*	2012b, 2014	9	76–78.8	3.4	8.3	−	0.8
	*PD_FP10*	2012b, 2014	10	114.4–121.2	3.7	8.9	−	0.4
	*PD_FP12.1*	2012b, 2014	12	41–44	3.6	8.6	−	0.4
	*PD_FP12.2*	2012a	12	41.3–44.1	6.8	18.2	−	0.7
	*PD_FP12.3*	2011	12	42.2–44.7	6.6	18.0	−	0.6

Based on the mapping results, five minor QTLs for FL were detected on chromosomes 2, 3, 5, and 9 in one environment. For FWd, four minor QTLs were identified on chromosomes 3, 4, and 7. All major and minor QTLs for FWg were detected on chromosome 7 at 29.6 to 30.7 cM, explaining more than 18.4% (LOD >7.5) of the phenotypic variation (R^2^) among four environments. For FP, three QTLs (*PD_FP9*, *PD_FP10*, *PD_FP12.1*) were commonly identified in two different environments but explained less than 10% of the phenotypic variation. However, the two remaining QTLs detected on chromosome 12 at 41.3 to 44.7 cM explained higher phenotype variation (>18%) in one environment. Except for *PD_FWd3*, all QTLs for FL, FWd, and FWg had positive additive effects, meaning that RILs with the maternal genotype had higher values, while all five QTLs for FP showed negative additive effects.

Using the same criteria as Han et al. (2016), QTLs detected in more than two environments with threshold R^2^ values of 10% were considered to be major QTLs. Only one QTL, *PD_FWg7.1*, was identified as a major QTL for FWg, with R^2^ (%) values ranging from 19.7 to 30.2. This large variation in R^2^ values indicates that FWg is highly affected by genotype × environment interactions in this population.

### Candidate Genes Influencing Major Fruit-Related Domestication Traits Under Selection

Based on the GWAS results, we selected 64 significant LD blocks and 230 SNPs related to the five major fruit-related domestication traits to predict candidate genes using Blast2GO. Among the 111 genes identified in the significant LD blocks, 1, 70, 39, and 16 genes were correlated with FL, FWd, FWg, PT, and FP, respectively, with some duplication (**Table S7**). Based on their predicted functions and communality for two or more closely related traits, 16 genes appeared to have close correlations with major fruit-related domestication traits.

First, a gene (*CA.PGA v.1.6.scaffold517.20*) located in the 211 Mb region of chromosome 4 in H04-0562 was strongly associated with FL. This gene, which is annotated as low-affinity sulfate transporter 3-like, is located approximately 1.7 kb from S04_211848210. A gene in the same mapping region at a 47 kb distance from SNP_211848210, *CA.PGA v.1.6.scaffold517.21*, is annotated as Agamous-like MADS-box protein AGL104; this gene appears to be an important regulator of FL (**Figures 3A, B**).

In the 227 Mb region of chromosome 4, a single gene, *CA.PGA v.1.6.scaffold1239.15*, was detected for both PT and FWg. This gene, encoding growth-regulating factor 1-like, and is be closely linked with SNP S04_227983120, a significant SNP located inside the 2nd exon (**Figures 3C–E** and **5A–C**). The varieties carrying the G allele had heavier fruits with thicker pericarps than varieties carrying the C allele (**Figures 3E** and **5C**).

*CA.PGAv.1.6.scaffold1368.1* (associated with LD block H12-0553) and *CA.PGAv.1.6.scaffold1387.3* (associated with LD block H12-0570) were predicted to be very important for FP due to their known associations with this trait. These two genes, which are physically positioned between 211 and 215 Mbp on chromosome 12, encode auxin-binding protein ABP19a-like and the protein BIG GRAIN 1-like A, respectively (**Figures 3F–H**).

Ten genes were closely related to FWd, including eight genes encoding various transcription factors and hormone-regulated genes on chromosome 9 (*CA.PGAv.1.6.scaffold3.11*, *CA.PGAv.1.6.scaffold3.10*, *CA.PGAv.1.6.scaffold5.32*, *CA.PGAv.1.6.scaffold5.22*, *CA.PGAv.1.6.scaffold5.16*, *CA.PGAv.1.6.scaffold5.14*, *CA.PGAv.1.6.scaffold283.11*, and *CA.PGAv.1.6.scaffold133.5*) and two genes on chromosome 12 (*CA.PGAv.1.6.scaffold730.39* and *CA.PGAv.1.6.scaffold534.6*) assembled in seven LD blocks (**Figure 4**). Among these, *CA.PGAv.1.6.scaffold3.10* and *CA.PGAv.1.6.scaffold5.16*, annotated as transcription repressor OFP12-like and leucine-rich repeat and IQ domain-containing protein 1-like isoform X3, respectively, are homologous to gene family members involved in domestication in tomato (OFP, IQ domain family) (**Figures 4B, D**). Additionally, two genes (*CA.PGAv.1.6.scaffold5.32* and *CA.PGAv.1.6.scaffold5.22*) associated with two stable SNPs in all three years of the experiment (S09_133144036, S09_136634573), which are annotated as elongation factor 1-beta-like and uncharacterized protein LOC107842678 isoform X1, respectively are also predicted to be important for FWd (**Figures 4C, D**). In addition, two genes (*CA.PGAv.1.6.scaffold5.14*, and *CA.PGAv.1.6.scaffold133.5*), which significant SNPs inside their coding regions, are annotated as mRNA cap guanine-N7 methyltransferase 1 and DNA-directed RNA polymerase II subunit 1, respectively (**Figures 4D, E, G, H**). Two SNPs (S09_138787607, S09_138787665) are located inside the 13th intron of *CA.PGAv.1.6.scaffold5.14*. Analysis of allelic frequency showed that plants with the T allele had wider fruits than plants with the C and G alleles (**Figure 4E**). Another SNP, S09_169434758, was located in the 4th exon of *CA.PGAv.1.6.scaffold133.5*. Among the 230 accessions, 186 accessions carrying the A allele had narrow fruits (average width of 17.95 mm), while 34 accessions carrying the G allele had relatively wide fruits (average width of 50.45 mm; **Figure 4H**).

Finally, four genes (*CA.PGAv.1.6.scaffold3.11*, *CA.PGAv.1.6.scaffold283.11*, *CA.PGAv.1.6.scaffold730.39*, and *CA.PGAv.1.6.scaffold534.6*) were closely related to significant SNPs commonly associated with FWg (**Figures 4B, F, J, K**).

Nine candidate genes are predicted to regulate FWg (**Figure 5**). Of these, *CA.PGAv.1.6.scaffold1239.15*, which regulates PT, as described above, is located on chromosome 4 and contains a significant SNP inside its coding region (**Figures 5B, C**). Moreover, *CA.PGAv.1.6.scaffold422.15*, which is annotated as peroxidase 41-like, is located 229.8 kb away from the significant SNP S06_194967541, which was consistently identified all three years of the experiment (**Figure 5E**). The seven remaining genes, which were commonly identified with FWd-associated genes, play roles in plant immunity and defense mechanisms (**Figures 5F–M**).

## Discussion

GWAS is often used to explore the genetic basis of complex traits in field-grown and horticultural crops due to its efficient detection of many natural allelic variations underlying phenotypic diversity (Brachi et al., 2011). Despite its successful use, however, it is still difficult to link the trait of interest to causal genes due to the widespread existence of population structure inside the diversity panels (Pritchard et al., 2000; Zhang et al., 2009). Population stratification and cryptic relationships can generate spurious associations between phenotypes and unlinked SNPs, leading to false positives (Ioannidis, 2005; Moonesinghe et al., 2007). In the current study, we identified 64 significant LD blocks linked to fruit-related traits and uncovered 16 candidate genes as major genes related to pepper domestication.

Pepper germplasm accessions have been divided into sub-clusters based on species, geographical origin, fruit characteristics, or different routes of introduction (Nicolai et al., 2013; Lee et al., 2016). Similar to previous reports, we identified four distinct sub-populations of pepper based on *Capsicum* species classification. To improve the reliability and credibility of the association results, we performed GWAS using only the *C. annuum* sub-cluster, which contains a large number of accessions, with great phenotypic variability but without any strong population stratification. We generated 187,966 genome-wide high-quality SNP markers from the *C. annuum* sub-cluster of the GWAS population using the GBS method. The LD blocks had an average size of 149 kb, indicating that at least 23,490 genome-wide SNPs are required for GWAS in pepper. Based on the estimated LD block size, the number SNP markers generated in this study is sufficient for GWAS in pepper.

We analyzed marker–trait associations for five major fruit-related domestication traits (FL, FWd, FWg, PT, FP) by GWAS. As a result, we identified 111 candidate genes within the 65 LD blocks. Of these, we selected 16 genes as strong candidate causal genes regulating fruit morphology according to the following criteria: 1) developmental genes known to be related to domestication in other plants; 2) genes within LD blocks containing significant SNPs detected in all three years of the study; and 3) SNP-containing genes associated with more than two traits.

Three genes (*CA.PGAv.1.6.scaffold517.21, CA.PGAv.1.6.scaffold3.10, CA.PGAv.1.6.scaffold5.16*), which are annotated as Agamous-like MADS-box protein AGL104, transcription repressor OFP12-like, and leucine-rich repeat and IQ domain-containing protein 1-like isoform X3, respectively, satisfied the first criterion, as they belong to the MADS domain subfamily, Ovate Family Protein (OFP) family, and IQ domain family, respectively. The OFP and IQ domain gene families include the well-known *ovate* and *sun* genes in tomato (Rodriguez et al., 2011). A nonsense mutation in the *ovate* gene is responsible for the development of pear-shaped fruit instead of oval-shaped fruit in tomato (Wang et al., 2007; Schmitz et al., 2015). In Arabidopsis, this gene regulates the production of a gibberellic acid (GA) biosynthesis enzyme to control cell elongation (Wang et al., 2007). AGAMOUS-like (AGL) transcription factors, which belong to the plant type I MADS domain subfamily, regulate reproductive development. A number of AGL transcription factor genes are specifically expressed in the central cell of the female gametophyte and endosperm in Arabidopsis (Bemer et al., 2010). Two genes associated with FP (*CA.PGAv.1.6.scaffold1368.1, CA.PGAv.1.6.scaffold1387.3*) are thought to be important candidates due to their regulation by auxin. The gene *CA.PGAv.1.6.scaffold1387.3*, which is annotated as BIG GRAIN 1-like A, is homologous to an auxin transport protein gene in Arabidopsis. This gene controls the adaxial–abaxial polarity of the pedicel (Yamaguchi et al., 2007), making it a good candidate gene for FP.

Five genes (*CA.PGAv.1.6.scaffold517.21, CA.PGAv.1.6.scaffold517.20, CA.PGAv.1.6.scaffold422.15, CA.PGAv.1.6.scaffold5.32*, and *CA.PGAv.1.6.scaffold5.22*) were chosen as candidate causal genes based on the second criterion: these genes are annotated as Agamous-like MADS-box protein AGL104, low-affinity sulfate transporter 3-like, peroxidase 41-like, elongation factor 1-beta-like, and uncharacterized protein LOC107842678, respectively. The first two genes, which are closely related to FL, are located at 211 Mbp on chromosome 4. In detail, *CA.PGAv.1.6.scaffold517.20*, a member of sulfate transporter family group 2, might be involved in the internal transport of sulfate between cellular or subcellular compartments within the plant (Hawkesford, 2003). Although sulfate is essential nutrient required for the biosynthesis of a wide range of sulfur-containing compounds, the functions of these genes in plants are unclear (Saito, 2000). The homolog of *CA.PGAv.1.6.scaffold422.15* (associated with FWg and located at 194 Mbp on chromosome 6) regulates pollen germination and pollen tube growth (Becker et al., 2003; Wang et al., 2008). The FWd-related genes include *CA.PGAv.1.6.scaffold5.22* and *CA.PGAv.1.6.scaffold5.32*. *CA.PGAv.1.6.scaffold5.32* is a homolog of Arabidopsis high amplitude circadian-regulating, which plays fundamental roles in nearly all aspects of plant growth and development (Covington et al., 2008). By contrast, the exact nature of the *CA.PGAv.1.6.scaffold5.22* gene homolog has yet to be characterized.

Seven genes (*CA.PGAv.1.6.scaffold3.11, CA.PGAv.1.6.scaffold3.10, CA.PGAv.1.6.scaffold5.32, CA.PGAv.1.6.scaffold5.22, CA.PGAv.1.6.scaffold283.11, CA.PGAv.1.6.scaffold730.39*, and *CA.PGAv.1.6.scaffold534.6*) were commonly identified as candidates for both FWd and FWg. Three of these genes (*CA.PGAv.1.6.scaffold3.11, CA.PGAv.1.6.scaffold730.39, CA.PGAv.1.6.scaffold534.6*) are closely related to plant immune responses and are annotated probable serine/threonine-protein kinase Cx32, probable LRR receptor-like serine/threonine-protein kinase At3g47570, and flower-specific defensin-like, respectively. Besides their major roles, a few studies have focused on their roles in plant growth and development (Chevalier and Walker, 2005; Schulz et al., 2013). *CA.PGAv.1.6.scaffold283.11*, annotated as calcium-dependent protein kinase 13, is homologous to an Arabidopsis gene encoding a transcriptional regulator essential for Nod-factor-induced gene expression in response to elevated calcium levels, which regulate secondary growth and biomass accumulation (Sehr et al., 2010).

*CA.PGAv.1.6.scaffold1239.15* encodes a Growth-regulating factor (GRF) that is correlated with both PT and FWg. GRFs are plant-specific transcription factors that were originally identified for their roles in stem and leaf development. Recent studies have highlighted their importance in other central developmental processes including flower and seed formation, root development, and the coordination of growth processes under adverse environmental conditions (Omidbakhshfard et al., 2015). We subjected the results of our phenotypic survey (conducted for three years to examine morphological traits in pepper) to Pearson correlation (r) analysis, which also supported the GWAS results.

A comparison of the QTLs mapped based on the PDRIL and GWAS results from the GWAS population revealed only one common genetic area associated with FP. Region 141.6 to 144.6 Mbp on chromosome 12 contains three QTLs (*PD_FP12.1*, *PD_FP12.2*, *PD_FP12.3*) and two significant SNPs (S12_143380249, S12_143380271). Inside this common area, two genes were identified (*CA.PGAv.1.6.scaffold18.1*, and *CA.PGAv.1.6.scaffold172.10*); these genes are annotated as L-ascorbate oxidase and ELKS/Rab6-interacting/CAST family member 1 isoform X1, respectively. Although the functional relevance of these candidate genes requires further validation, based on their putative functions, they represent strong candidate genes involved in pepper domestication. Among the 17 detected QTLs, one major QTL for FWg, *PD_FWg7.1*, spanning around 68.9 to 73.6 Mbp on chromosome 7 was identified. In this position, we were able to detect a relatively high peak than the surrounding area in GWAS. However, the P-values of those SNPs (–log10 P-value <2.9) did not pass a significant threshold. Some QTL positions for FWg and FWd were corresponding to QTLs reported by Wu et al. (2019). Unexpectedly, however, most QTLs or significant SNPs in QTL analysis and GWAS for fruit traits were not common. This may be due to several reasons including Beavis effect, differences in models or fundamental differences analysis as suggested Hansson et al. (2018).

In summary, we successfully used GWAS to identify genes responsible for major fruit-related traits in pepper. The significant haplotypes identified in this study provide unique molecular footprints for developing markers for pre-breeding or genomic selection. Future functional validation of the candidate genes identified in this study should provide additional targets for the improvement of major horticultural traits in pepper *via* breeding.

## Data Availability Statement

The datasets generated for this study can be found in The National Agricultural Biotechnology Information Center http://nabic.rda.go.kr/, ID NV-0630-000001.

## Author Contributions

Conceptualization: H-YL, B-CK. Data curation: H-YL, N-YR. Formal analysis: H-YL. Funding acquisition: B-CK, J-KK. Investigation: H-YL, N-YR. Methodology: H-YL. Project administration: H-YL, B-CK. Resources: B-CK, N-YR. Software: H-YL, J-HL. Validation: B-CK. Visualization: H-YL. Writing—original draft: H-YL. Writing—review and editing: B-CK, AP.

## Funding

This work was carried out with the support of “Cooperative Research Program for Agriculture Science & Technology Development (Project No. PJ01322901)” Rural Development Administration, Republic of Korea.

## Conflict of Interest

The authors declare that the research was conducted in the absence of any commercial or financial relationships that could be construed as a potential conflict of interest.
