# Supplementary figures and images for: Uncovering Candidate Genes Controlling Major Fruit-Related Traits in Pepper via Genotype-by-Sequencing Based QTL Mapping and Genome-Wide Association Study
Source: Front Plant Sci. 2020 Jul 23;11:1100. doi: 10.3389/fpls.2020.01100 (PMC7390901; doi:10.3389/fpls.2020.01100)

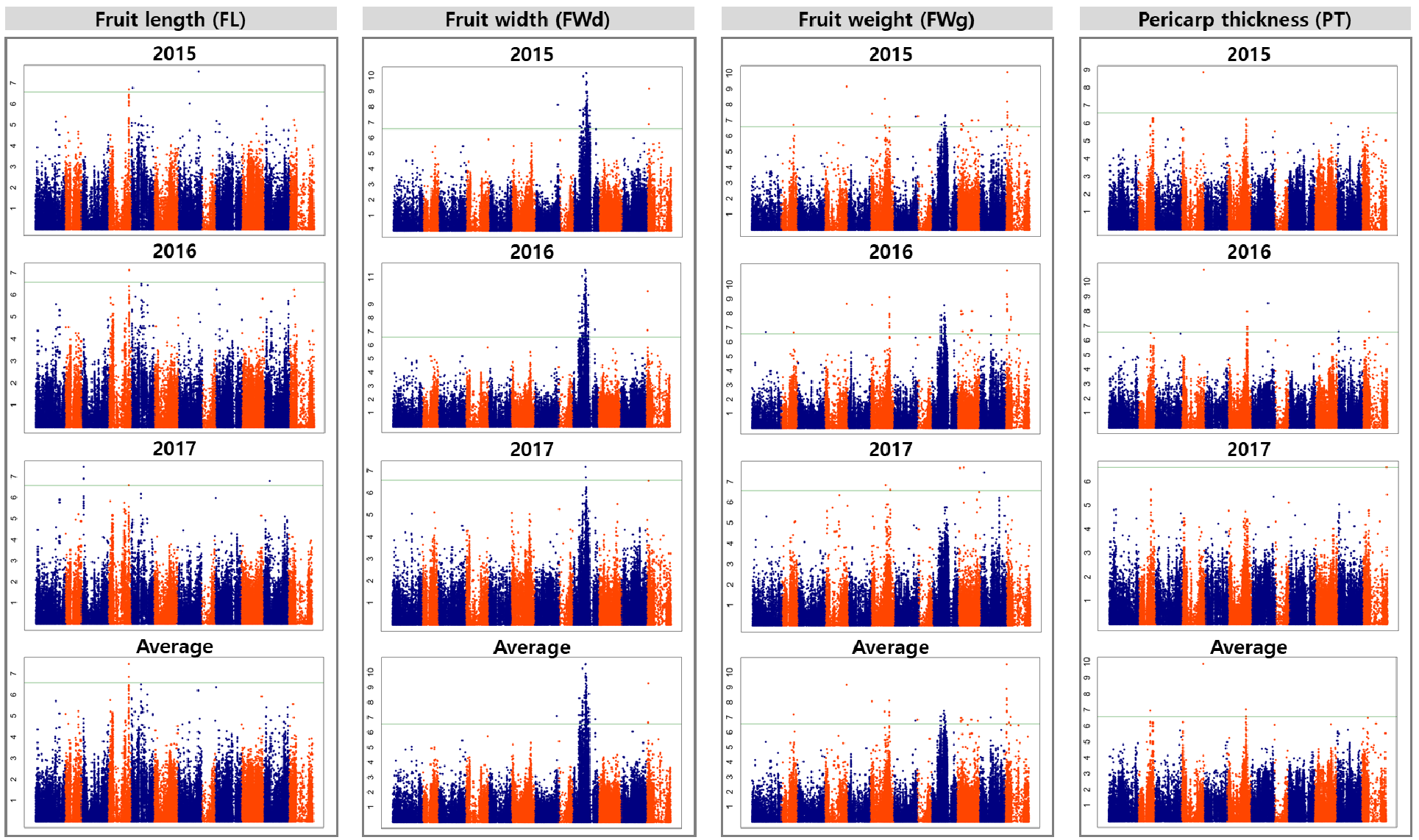

Supplement: Supplementary file 1 [file Image_1.tiff]

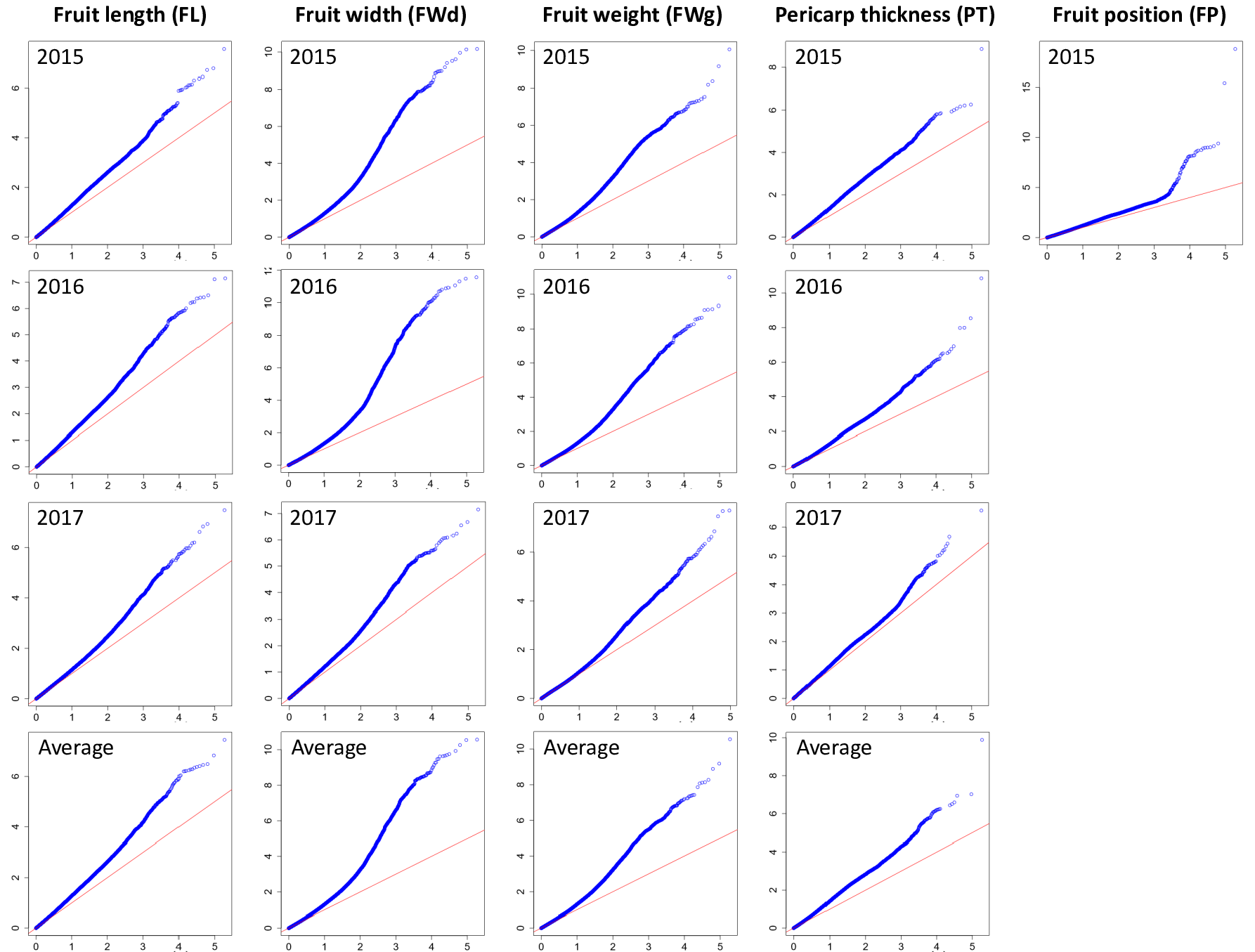

Supplement: Supplementary file 2 [file Image_2.tiff]

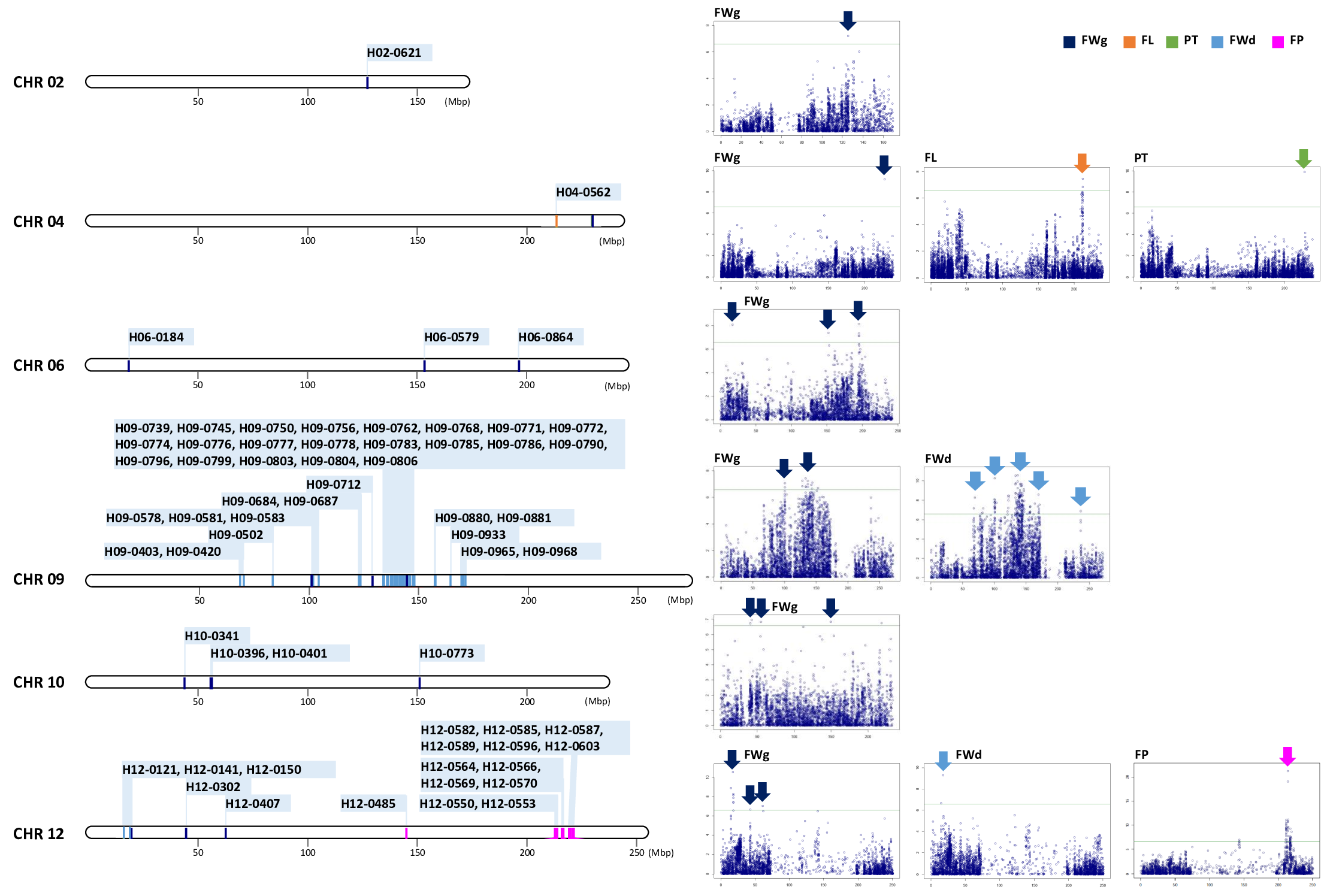

Supplement: Supplementary file 3 [file Image_3.tiff]

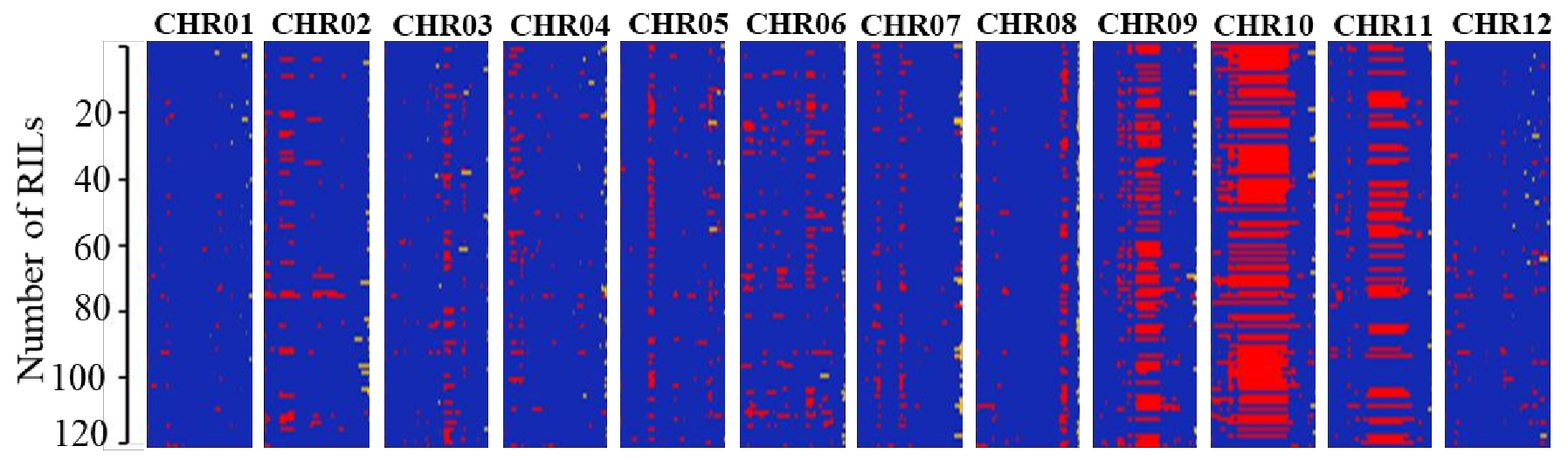

Supplement: Supplementary file 4 [file Image_4.tiff]
